# Changes in structure and function of social networks of independently living middle-aged and older adults in diverse sociodemographic subgroups during the COVID-19 pandemic: a longitudinal study

**DOI:** 10.1186/s12889-022-14500-2

**Published:** 2022-12-03

**Authors:** Lisanne CJ Steijvers, Stephanie Brinkhues, Theo G van Tilburg, Christian JPA Hoebe, Mandy MN Stijnen, Nanne de Vries, Rik Crutzen, Nicole HTM Dukers-Muijrers

**Affiliations:** 1 Department of Social Medicine, Care and Public Health Research Institute (CAPHRI), Maastricht University, Maastricht, the Netherlands; 2 Department of Sexual Health, Infectious Diseases, and Environmental Health, Public Health Service South Limburg, Heerlen, the Netherlands; 3 Department of Knowledge and Innovation, Public Health Service South Limburg, Heerlen, the Netherlands; 4 Department of Sociology, Vrije Universiteit Amsterdam, Amsterdam, the Netherlands; 5 Department of Medical Microbiology, Care and Public Health Research Institute (CAPHRI), Maastricht University Medical Center (MUMC+), Maastricht, the Netherlands; 6 Department of Health Services Research, Care and Public Health Research Institute (CAPHRI), Maastricht University, Maastricht, the Netherlands; 7 Department of Health Promotion, Care and Public Health Research Institute (CAPHRI), Maastricht University, Maastricht, the Netherlands

**Keywords:** Social networks, Network size, Social support, Sociodemographic subgroups

## Abstract

**Background:**

Social networks, i.e., all social relationships that people have, contribute to well-being and health. Governmental measures against COVID-19 were explicitly aimed to decrease physical social contact. We evaluated ego-centric social network structure and function, and changes therein, among various sociodemographic subgroups before and during the COVID-19 pandemic.

**Methods:**

Independently living Dutch adults aged 40 years and older participating in the SaNAE longitudinal cohort study filled in online questionnaires in 2019 and 2020. Changes in network size (network structure) and social supporters (network function) were assessed. Associations with risk for changes (versus stable) were assessed for sociodemographic subgroups (sex, age, educational level, and urbanization level) using multivariable regression analyses, adjusted for confounders.

**Results:**

Of 3,344 respondents 55% were men with a mean age of 65 years (age range 41–95 in 2020). In all assessed sociodemographic subgroups, decreases were observed in mean network size (total population: 11.4 to 9.8), the number of emotional supporters (7.2 to 6.1), and practical supporters (2.2 to 1.8), and an increase in the number of informational supporters (4.1 to 4.7). In all subgroups, the networks changed to being more family oriented. Some individuals increased their network size or number of supporters; they were more often women, higher-educated, or living in rural areas.

**Conclusion:**

The COVID-19 pandemic impacted social networks of people aged 40 years and older, as they increased informational support and reduced the number of their social relationships, mainly in terms of emotional and practical supporters. Notably, some individuals did not show such unfavorable trends and managed to reorganize their networks to attribute social support roles more centrally.

**Supplementary Information:**

The online version contains supplementary material available at 10.1186/s12889-022-14500-2.

## Background

Society at large and especially sociodemographic subgroups were severely impacted by both the pandemic as social distancing measures (keep physical distance from others, restrict the number of contacts, and avoid crowded places [1]). Several studies in Europe and the United States have shown that number of contacts in the population has drastically decreased during the COVID-19 pandemic [2–5]. Even though social contact poses a risk for transmission, connecting with others also is a core human need and a causal factor for good health [6, 7]. Social distancing may have led to decreasing health benefits from social relations, causing a major negative impact on the health of middle-aged and older adults [8]. A vast body of evidence demonstrates that having fewer social relationships is associated with a higher risk of all-cause mortality, the onset of cardiovascular diseases, and Type 2 Diabetes Mellitus (T2DM) in individuals of middle and older age, and with higher inflammation levels among cancer patients and T2DM complications [9–11].

Social networks, i.e., all social relationships that people have, may generate social support, such as informational support (receiving information), emotional support (discussing important topics), and practical (instrumental) support, that helps to gain and maintain health, cope with disease, and affect physical and mental resilience [12, 13]. Social support is about social network functioning, and typifies how to participate in society and how to harvest social relationships for gaining access to health benefits, support, and resources [14]. Network size, the number of social relationships, is a structural network aspect. Other structural network aspects include the type of relationships, such as family, friends, neighbors, and colleagues (diversity) [15]. Structural and functional social network aspects are intertwined. The type of relationship is important for the type of social support provided. Where close family members and friends (“stronger ties”) often provide emotional support, more distant relationships (also called “weaker ties”) are often important for the provision of informational support (especially new information) [16].

Social networks are shaped by socio-cultural conditions [15, 17]. Sex differences in social networks have already been established in multiple studies. Men tend to have smaller, less diverse networks with less social supporters than women, because men provide support to or receive support from their spouses, whereas women often provide support to but also receive social support from family members and friends [15]. Older persons also tend to have smaller, less diverse networks, but receive more social support, which could be explained by several factors [18]. One of these factors is the socioemotional selectivity theory. This theory proposes that older persons focus more on investing emotionally in existing social relationships instead of gaining new relationships [19]. In addition to sex and age differences in social networks, there also have been educational level differences assessed. Lower educated persons tend to have smaller and family-centered social networks compared to higher educated persons [17, 20].

People’s social networks, i.e., structural, and functional social network aspects, have changed during the COVID-19 pandemic. Several studies have assessed changes in daily contact frequency during the pandemic, reporting a decrease in the number of social network members who were daily contacted in-person [2–5]. Furthermore, a Dutch panel study assessed changes in social networks of adults aged 18 to 35 years, and 65 years of age during first lockdown of the COVID-19 pandemic [21]. A decrease in the number of network members, and a shift towards stronger ties being attributed more centrally within the network was established [21]. Social network size was measured by asking with whom important topics are discussed (e.g., emotional supporters) and who helps with jobs around the house (e.g., practical support).

Völker established changes in social networks during the first wave of the COVID-19 pandemic [21]. However, the COVID-19 pandemic has once again emphasized health inequalities among various sociodemographic groups [22–29]. Inequalities in social networks [15, 17, 18, 20, 30] and changes in social networks pre-COVID time have already been established [31]. Therefore, sociodemographic subgroups might experience a “double burden”, highlighting the importance of generating novel insights into the societal impact of the COVID-19 pandemic. Knowledge about changes in social network structure and function, and how these changes differ among various subgroups could provide lead to social network formation and activation in future pandemics.

The Social Network Assessment in Adults and Elderly (SaNAE) study aims to gain insight into the composition of social networks in relation to health for independently living Dutch adults aged 40 years and older. The current report explores how structural and functional social network aspects have changed in 2020 compared to the pre-COVID time in 2019 among sociodemographic subgroups. We hypothesized that network size and the number of social supporters are negatively impacted by the COVID-19 pandemic, especially among the more vulnerable people, who -pre-COVID time- started with less developed network sizes.

## Methods

### Ethics statement

This study was approved by the Medical Ethical Committee of the University of Maastricht (METC 2018 − 0698, 2019 − 1035, and 2020–2266). Before starting the questionnaires, both baseline in 2019 and follow-up in 2020, participants first gave electronic informed consent. All methods were carried out following relevant guidelines and regulations.

### Study design

This longitudinal study used data from the SaNAE study (www.sanae-study.nl) and is reported according to the STROBE guidelines [32]. The baseline measurement was conducted between March and April 2019, and the follow-up measurement in August and November 2020 was in between two lockdown periods of the COVID-19 pandemic.

### Study population

**Fig. 1:**
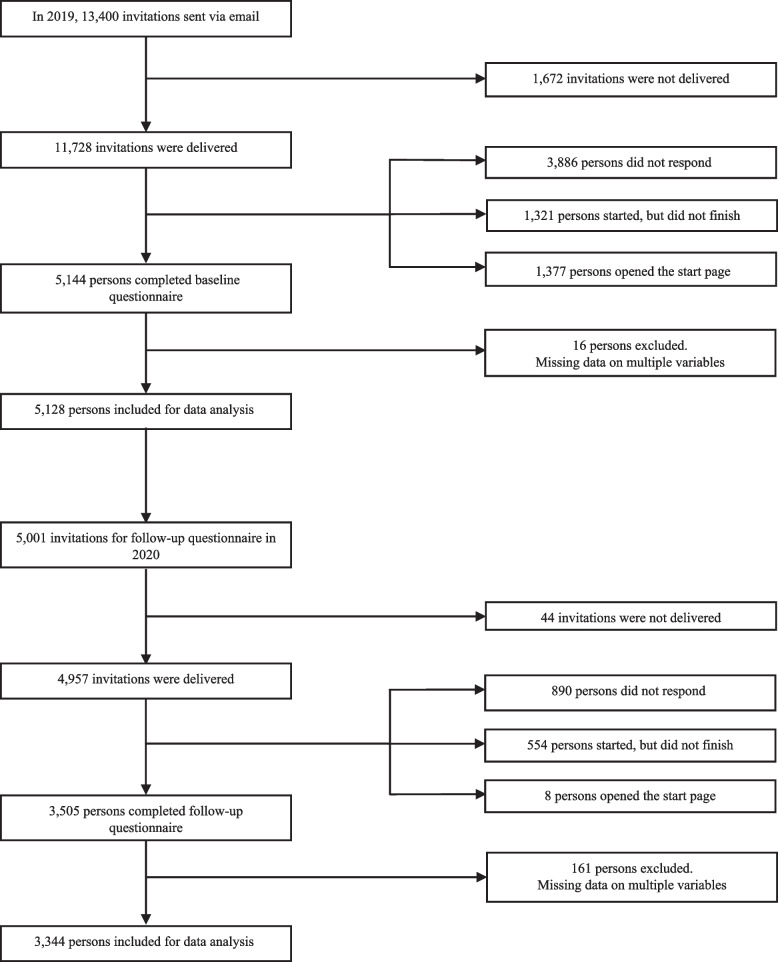
Flowchart of people aged 40 years and older in the Netherlands (SaNAE study population)

Inclusion criteria for participants were being 40 years of age or older and living independently in the Dutch province of Limburg. For the first measurement, 11,728 persons were invited via email. The email provided a link to the online questionnaire. Invitees previously participated in the ‘Dutch Health Monitor’, which is a population-based questionnaire of the Public Health Services South and North Limburg [33]. In total 5,144 persons (44%) responded, of whom 5,001 (97%) also provided consent to be invited for further cohort questionnaires. Of these invitees, 67% (*n* = 3,505) participated in 2020 and of those 94% (*n* = 3,344) provided complete questionnaire data. 2019 participants who were lost to follow-up were slightly younger compared to 2020 participants (mean difference 1.8 years, *p* < 0.001), but did not differ in network size, or the number of supporters (*p* > 0.05). Only participants with no missing data on either dependent or independent variables were included in analyses (*n* = 3,344) (Fig. 1).

### Measurements

The participants’ age was divided into four categories: 40–49, 50–59, 60–69, and 70 years and older. Educational level was categorized into lower (no education, primary education (not completed), and lower vocational education), medium (intermediate vocational and higher secondary education), and higher educational level (higher professional and university education). The level of urbanization was based on address density derived from the respondents’ postal code, in five categories: rural, hardly, moderately, strongly, and extremely urbanized areas [34]. Due to the small numbers in the last category, categories of strongly and extremely urbanized areas were merged.

Social networks were assessed using a name generator questionnaire [35]. In brief, the name generator questionnaire asks participants to fill in the names of family members, friends, acquaintances, and other persons who currently are important to them or provide support. For each identified network member, additional information was asked using name interpreter items (Supplementary file 1).

### Structural social network aspects

Network size was the number of network members identified by a respondent. Respondents were able to name up to fifteen family members, ten friends, ten acquaintances (i.e., neighbors, colleagues, club members), and five other members, resulting in a maximum network size of forty persons. The share of a specific type of relationship was computed as a percentage with the denominator being the network size. This percentage reflected the distribution of relationship types within the given network.

### Functional social network aspects

Informational social support was the number of network members who advised on problems. Emotional social support was defined as the number of network members who provided the opportunity to discuss important matters or provided support if one feels unwell. Practical social support was the number of network members who helped with small or larger tasks around the house. The number of network members who provided support was referred to as “the number of supporters”. Alongside the number of supporters, also the share of a specific type of supporters (informational, emotional, practical) by percentage was computed with network size as the denominator. The share (i.e., the percentage) thereby reflected the composition of relationship structure and function within the given network.

### Changes in social network aspects

Individual changes in the network were computed by subtracting computed structural network measures (number and percentage) of 2019 from that of 2020. Furthermore, for network size and the number of supporters, additional variables were constructed, allowing individual variation in the change in network size and the number of supporters. The three categories for network size change reflected whether the individual change was a decrease (by two members or more), an increase (of two members or more), or stable (no change or only one member less or more). Similar variables were constructed to reflect a change in the number of supporters (by one member or more).

### Statistical analyses

Paired sample *t*-tests were used to test distributions in social network aspects (number and percentage) of the study population over time (2020 versus 2019). The mean change in numbers of network members and social supporters, and percentage point of shares of the network member-type, and social supporters were presented for the network aspects. Furthermore, the grouped average changes in network size, number of supporters, and percentage point shares of the network member types by sociodemographic factors were tested using paired sample *t*-tests. Then, we explored sociodemographic determinants (i.e., sex, age, educational level, and urbanization) for their association with the main network aspects (network size, informational, emotional, and practical support) and network member types as reported in 2020 by performing linear regression analyses. Lastly, we assessed whether sociodemographic determinants were associated with changes in the number of network members or the number of supporters, using mean change variables in two categories (decrease versus stable or increase versus stable). Multivariable logistic regression analyses were performed for decrease and increase in the number of network members and the number of supporters separately, with the sociodemographic determinants as independent variables. For descriptive purposes, several reference categories for independent variables were inverted based on effect size. All analyses were adjusted for network size and living arrangement as reported in 2019, as the number of network members and living alone at baseline predict changes in network size and supporters in 2020. A *p*-value < 0.05 indicated statistical significance. All analyses were performed using IBM SPSS Statistics (version 26.0).

## Results

### Study population

In 2020, 55% of the study population were men and the mean age of respondents was 65 years (range 41 to 95 years) (Table 1). Overall, 43% of the population had a higher educational level, up to a quarter lived in a rural area, and 20% lived in a moderately urbanized area.

**Table 1:** Demographic characteristics of people aged 40 years and older in the Netherlands (SaNAE study population) in 2020 (*n* = 3,344)

	% (n)
**Sex**	
Male	55 (1850)
Female	45 (1494)
**Age**	
40–49 years	8 (262)
50–59 years	19 (645)
60–69 years	38 (1280)
70 years and older	35 (1157)
**Educational level**	
Low (no education, completed or not completed primary education, and lower vocational education)	27 (887)
Medium (intermediate vocational and higher secondary education)	30 (1008)
High (higher professional and university education)	43 (1449)
**Urbanization**	
Rural areas (< 500 addresses per km^2^)	27 (904)
Hardly urbanized areas (500 to 1000 addresses per km^2^)	24 (788)
Moderately urbanized areas (1000–1500 addresses per km^2^)	19 (636)
Strongly or extremely urbanized areas (> 1500 addresses per km^2^)	30 (1016)

### Structural social network characteristics

#### Changes in network size in sociodemographic subgroups

Changes in network size in various sociodemographic factors are shown in Table 2. In short, mean network size decreased in all sociodemographic subgroups, though the decrease was most prominent in people who were older, low educated, or living in urban areas.

**Table 2:** Social network size and social support in 2019 and 2020 by sociodemographic subgroups

	Network size			Number of informational supporters			Number of emotional supporters			Number of practical supporters		
	**2019**	**2020**	**Change**	**2019**	**2020**	**Change**	**2019**	**2020**	**Change**	**2019**	**2020**	**Change**
	**Mean (sd)**	**Mean (sd)**	**Mean (sd)**	**Mean (sd)**	**Mean (sd)**	**Mean (sd)**	**Mean (sd)**	**Mean (sd)**	**Mean (sd)**	**Mean (sd)**	**Mean (sd)**	**Mean (sd)**
Sex												
Male	10.0 (7.6)	8.5 (6.7)	-1.6 (6.6)^***^	3.5 (4.1)	4.0 (4.5)	0.4 (4.6)^***^	6.1 (5.7)	5.2 (5.2)	-0.9 (5.6)^***^	2.2 (2.6)	1.7 (2.1)	-0.5 (2.8)^***^
Female	13.0 (8.0)	11.6 (7.4)	-1.5 (6.6)^***^	4.8 (4.3)	5.7 (5.5)	0.8 (5.0)^***^	8.4 (6.3)	7.2 (5.8)	-1.2 (5.5)^***^	2.1 (2.2)	1.9 (2.0)	-0.2 (2.3)^***^
Age												
40–49 years	11.3 (7.5)	10.7 (7.3)	-0.6 (5.9)	5.3 (4.7)	6.1 (6.3)	0.7 (5.0)^*^	7.6 (6.1)	6.8 (6.2)	-0.7 (5.1)^*^	2.6 (2.6)	2.1 (1.9)	-0.5 (2.6)^**^
50–59 years	11.6 (7.7)	10.5 (6.9)	-1.1 (5.9)^***^	4.7 (4.5)	5.6 (5.2)	1.0 (4.9)^***^	7.6 (6.2)	6.8 (5.4)	-0.9 (5.5)^***^	2.3 (2.5)	1.9 (1.8)	-0.4 (2.6)^***^
60–69 years	11.7 (8.2)	10.3 (7.7)	-1.4 (6.9)^***^	4.0 (4.1)	4.8 (5.2)	0.8 (4.9)^***^	7.5 (6.4)	6.4 (5.9)	-1.1 (5.8)^***^	2.1 (2.4)	1.7 (2.0)	-0.4 (2.5)^***^
70 years and older	10.9 (7.8)	8.8 (6.7)	-2.1 (6.8)^***^	3.6 (4.0)	3.8 (4.1)	0.2 (4.5)	6.4 (5.5)	5.3 (5.1)	-1.1 (5.4)^***^	2.1 (2.5)	1.8 (2.2)	-0.3 (2.7)^***^
Educational level												
Low	9.5 (7.1)	7.8 (6.1)	-1.7 (6.1)^***^	3.3 (3.6)	3.6 (4.1)	0.3 (4.4)^**^	5.4 (4.8)	4.6 (4.5)	-0.9 (4.8)^***^	1.9 (2.2)	1.7 (2.1)	-0.2 (2.4)^**^
Medium	11.2 (7.8)	9.8 (7.2)	-1.5 (6.6)^***^	4.1 (4.2)	4.8 (5.0)	0.8 (4.9)^***^	6.8 (5.7)	6.2 (5.6)	-0.6 (5.5)^***^	2.2 (2.5)	1.8 (1.8)	-0.4 (2.6)^***^
High	12.6 (8.2)	11.2 (7.6)	-1.4 (6.9)^***^	4.6 (4.5)	5.3 (5.4)	0.7 (4.9)^***^	8.4 (6.7)	7.0 (6.0)	-1.4 (6.0)^***^	2.3 (2.5)	1.9 (2.2)	-0.4 (2.7)^***^
Urbanization												
Rural areas	12.2 (8.4)	10.9 (7.6)	-1.3 (7.0)^***^	4.5 (4.4)	5.4 (5.4)	0.9 (4.9)^***^	7.9 (6.5)	6.8 (6.0)	-1.1 (5.9)^***^	2.3 (2.5)	2.0 (2.2)	-0.3 (2.6)^***^
Hardly urbanized areas	11.7 (8.0)	10.0 (7.4)	-1.7 (6.7)^***^	4.1 (4.1)	4.6 (5.0)	0.4 (4.7)^**^	7.3 (6.2)	6.2 (5.5)	-1.1 (5.7)^***^	2.2 (2.5)	1.7 (1.9)	-0.6 (2.7)^***^
Moderately urbanized areas	10.8 (7.5)	9.2 (7.0)	-1.6 (6.3)^***^	3.9 (4.4)	4.4 (5.0)	0.5 (4.9)^*^	6.8 (5.6)	5.7 (5.4)	-1.1 (5.4)^***^	2.2 (2.7)	1.8 (2.2)	-0.4 (2.8)^**^
Strongly or extremely urbanized areas	10.7 (7.6)	9.1 (6.8)	-1.6 (6.3)^***^	3.9 (4.0)	4.5 (4.6)	0.6 (4.5)^***^	6.7 (5.8)	5.7 (5.4)	-0.9 (5.3)^***^	2.0 (2.2)	1.7 (2.1)	-0.3 (2.4)^***^
Living alone	10.2 (7.3)	9.0 (6.6)	-1.2 (6.0)^***^	3.6 (3.9)	4.1 (4.4)	0.5 (4.1)^**^	6.3 (5.4)	5.5 (5.0)	-0.8 (4.7)^***^	1.8 (2.2)	1.6 (2.2)	-0.2 (2.4)^*^

*Sd* Standard deviation

^*^p < 0.05,

^**^p < 0.01,

^***^p < 0.001

Focusing on the network in 2020, some subgroups had smaller networks. Men, lower and medium educated persons, and people living in moderately, strongly, or extremely urbanized areas had smaller network sizes in 2020 compared to their counterparts (Table 3).

**Table 3:** Multivariable linear regression of social network characteristics on sociodemographic characteristics in 2020

	Network size		Informational supporters		Number of emotional supporters		Number of practical supporters	
	**B (S.E.)**	***p***	**B (S.E.)**	***p***	**B (S.E.)**	***p***	**B (S.E.)**	***p***
**(Constant)**	7.565 (0.518)	< 0.001	4.409 (0.410)	< 0.001	4.671 (0.440)	< 0.001	1.118 (0.183)	< 0.001
** Sex**								
Male	-1.418 (0.203)	< 0.001	-0.659 (0.160)	< 0.001	-0.898 (0.172)	< 0.001	0.130 (0.072)	0.070
Female	ref		ref		ref		ref	
** Age**								
40–49 years	ref		ref		ref		ref	
50–59 years	-0.055 (0.408)	0.892	-0.405 (0.322)	0.209	-0.060 (0.346)	0.859	-0.272 (0.144)	0.059
60–69 years	-0.112 (0.381)	0.768	-1.105 (0.301)	< 0.001	-0.200 (0.323)	0.231	-0.483 (0.134)	< 0.001
70 years and older	-0.926 (0.390)	0.018	-1.797 (0.309)	< 0.001	-0.829 (0.331)	0.007	-0.384 (0.138)	0.005
** Educational level**								
Low	-1.727 (0.244)	< 0.001	-0.694 (0.193)	< 0.001	-1.318 (0.207)	< 0.001	0.113 (0.086)	0.187
Medium	-0.842 (0.231)	< 0.001	-0.310 (0.182)	0.089	-0.478 (0.196)	0.015	-0.020 (0.081)	0.802
High	ref		ref		ref		ref	
** Urbanization**								
Rural areas	ref		ref		ref		ref	
Hardly urbanized areas	-0.454 (0.271)	0.094	-0.585 (0.214)	0.006	-0.237 (0.230)	0.303	-0.257 (0.096)	0.007
Moderately urbanized areas	-0.767 (0.288)	0.008	-0.509 (0.228)	0.026	-0.448 (0.245)	0.067	-0.033 (0.102)	0.744
Strongly or extremely urbanized areas	-0.790 (0.255)	0.002	-0.449 (0.202)	0.026	-0.342 (0.216)	0.114	-0.165 (0.090)	0.066

In all sociodemographic subgroups, within their social networks in 2020, the distribution of relationship types had shifted to a higher percentage being family members (Table 4).

**Table 4:** Social network composition (proportion relationship types) by sociodemographic characteristics

	Proportion family members			Proportion friends			Proportion acquaintances			Proportion extra members		
	**2019**	**2020**	**Change**	**2019**	**2020**	**Change**	**2019**	**2020**	**Change**	**2019**	**2020**	**Change**
	**Mean (sd)**	**Mean (sd)**	**Mean (sd)**	**Mean (sd)**	**Mean (sd)**	**Mean (sd)**	**Mean (sd)**	**Mean (sd)**	**Mean (sd)**	**Mean (sd)**	**Mean (sd)**	**Mean (sd)**
**Sex**												
Male	50.0 (25.7)	56.8 (27.5)	6.8 (29.4)^***^	24.8 (19.2)	22.9 (20.0)	-1.8 (21.7)^***^	17.5 (16.4)	15.7 (16.7)	-1.8 (19.7)^***^	7.7 (13.6)	4.6 (12.1)	-3.2 (17.7)^***^
Female	43.3 (20.1)	48.6 (22.6)	5.3 (22.6)^***^	29.8 (16.1)	28.5 (17.0)	-1.3 (18.1)^**^	19.3 (14.1)	17.4 (14.4)	-2.0 (16.4)^***^	7.6 (11.3)	5.5 (11.6)	-2.1 (15.8)^***^
**Age**												
40–49 years	46.8 (22.3)	53.4 (24.6)	6.6 (25.0)^***^	27.4 (17.3)	26.3 (17.5)	-1.2 (16.3)	19.8 (15.5)	17.2 (15.3)	-2.6 (17.4)^*^	6.0 (12.8)	3.1 (9.9)	-2.9 (15.5)^**^
50–59 years	45.0 (22.0)	51.8 (24.4)	6.7 (24.2)^***^	27.3 (16.5)	25.2 (17.6)	-2.0 (17.1)^**^	20.4 (16.1)	18.7 (15.7)	-1.6 (18.4)^*^	7.4 (12.1)	4.3 (9.6)	-3.1 (14.8)^***^
60–69 years	46.7 (23.2)	53.0 (26.6)	6.3 (25.8)^***^	27.2 (18.0)	26.2 (19.5)	-1.0 (20.2)	18.2 (14.9)	15.8 (15.7)	-2.4 (17.6)^***^	7.9 (12.6)	5.0 (11.6)	-2.9 (16.8)^***^
70 years and older	48.3 (24.9)	53.8 (26.8)	5.5 (28.9)^***^	26.6 (19.0)	24.7 (19.3)	-2.0 (22.3)^**^	16.9 (15.4)	15.7 (15.9)	-1.2 (19.2)^*^	8.1 (12.9)	5.9 (13.6)	-2.3 (18.3)^***^
**Education**												
Low	51.1 (25.5)	57.1 (27.5)	6.0 (29.9)^***^	25.0 (19.1)	23.1 (20.1)	-1.9 (21.5)^**^	15.6 (15.6)	14.3 (16.3)	-1.3 (19.4)	8.3 (14.3)	5.5 (13.3)	-2.8 (19.3)^***^
Medium	47.5 (23.2)	53.8 (25.4)	6.2 (25.5)^***^	26.9 (18.2)	25.5 (19.0)	-1.4 (19.8)^*^	18.1 (15.7)	16.0 (15.4)	-2.1 (18.1)^***^	7.5 (12.7)	4.7 (11.4)	-2.8 (17.1)^***^
High	44.1 (22.1)	50.2 (24.5)	6.1 (25.2)^***^	28.3 (17.1)	26.8 (18.0)	-1.5 (19.5)^**^	20.1 (14.8)	18.0 (15.5)	-2.1 (17.8)^***^	7.6 (11.5)	5.0 (11.3)	-2.6 (15.1)^***^
**Urbanization**												
Rural areas	46.7 (22.9)	52.6 (24.7)	6.0 (25.7)^***^	26.4 (17.6)	24.9 (17.2)	-1.5 (19.1)^*^	19.2 (14.9)	17.6 (15.5)	-1.6 (17.7)^**^	7.8 (12.3)	4.9 (11.1)	-2.9 (16.2)^***^
Hardly urbanized areas	47.4 (22.2)	52.6 (25.3)	5.3 (24.3)^***^	26.7 (16.9)	26.0 (18.8)	-0.7 (19.3)	18.9 (15.1)	15.9 (15.3)	-2.9 (17.4)^***^	7.1 (11.3)	5.4 (11.7)	-1.7 (15.5)^**^
Moderately urbanized areas	47.5 (24.3)	53.3 (26.7)	5.8 (27.7)^***^	27.0 (17.9)	25.4 (19.9)	-1.7 (20.5)^*^	17.1 (15.7)	15.7 (15.8)	-1.5 (19.2)	8.4 (13.5)	5.7 (14.1)	-2.6 (19.6)^***^
Strongly or extremely urbanized areas	46.6 (24.7)	53.7 (26.5)	7.2 (28.3)^***^	27.9 (19.3)	25.6 (19.8)	-2.3 (21.4)^***^	17.8 (15.9)	16.3 (16.3)	-1.5 (18.9)^*^	7.7 (13.3)	4.4 (11.1)	-3.4 (16.7)^***^

*Sd* Standard deviation

^*^p < 0.05,

^**^p < 0.01,

^***^p < 0.001

#### Individual level changes in network size

While mean network size decreased in all sociodemographic subgroups, individual variation was observed. Assessing change in individual persons, it was observed that in 46% (*n* = 1,545) of respondents, network size decreased (mean decrease of 7 members, range − 2 to -33), in 26% of the respondents (*n* = 861) network size was unchanged and in 28% of the respondents (*n* = 938) network size increased (mean increase of 6, range 2 to 32). Compared to having an unchanged network size, to have decreased this number was more likely when being a man, older, lower educated, or living in moderately, strongly, and extremely urbanized areas; to have increased network size was more likely when being a woman or higher educated (Table 5). All odds ratios ranged between 1.18 and 1.57.

**Table 5:** Multivariable logistic regression of decreases versus stable and increases versus stable in network size and social support by sociodemographic characteristics

	Decreased numbers		Stable (reference)	Increased numbers	
	**% (n)**	**OR (95% CI)** ^**a**^	**% (n)**	**% (n)**	**OR (95% CI)** ^**a**^
**Network size**					
** Sex**					
Male	45.8 (847)	**1.31 (1.08–1.61)** ^**^	27.2 (503)	27.0 (500)	Ref
Female	46.7 (698)	Ref	24.0 (358)	29.3 (438)	**1.25 (1.02–1.53)** ^*^
** Age**					
40–49 years	42.3 (130)	1.39 (0.92–2.09)	23.8 (73)	33.9 (104)	1.31 (0.90–1.90)
50–59 years	40.5 (287)	Ref	27.7 (196)	31.8 (225)	1.01 (0.77–1.33)
60–69 years	47.1 (611)	1.20 (0.92–1.56)	25.9 (336)	26.9 (349)	0.98 (0.78–1.22)
70 years and older	50.9 (438)	**1.57 (1.19–2.06)** ^**^	24.4 (210)	24.7 (212)	Ref
** Educational level**					
Lower	47.1 (418)	**1.36 (1.08–1.72)** ^*^	28.4 (252)	24.5 (217)	Ref
Medium	44.7 (451)	1.06 (0.85–1.33)	27.5 (277)	27.8 (280)	1.13 (0.88–1.46)
Higher	46.7 (676)	Ref	22.9 (332)	30.4 (441)	**1.50 (1.18–1.91)** ^***^
** Urbanization**					
Rural areas	42.7 (386)	Ref	25.6 (231)	31.7 (287)	Ref
Hardly urbanized areas	47.3 (372)	1.20 (0.92–1.57)	25.4 (200)	27.4 (216)	0.91 (0.70–1.18)
Moderately urbanized areas	49.1 (312)	**1.45 (1.09–1.92)** ^*^	25.0 (159)	25.9 (165)	0.89 (0.67–1.17)
Strongly or extremely urbanized areas	47.4 (400)	**1.34 (1.04–1.72)** ^*^	25.6 (216)	27.0 (228)	0.84 (0.66–1.08)
**Number of informational supporters**					
** Sex**					
Male	35.8 (662)	0.82 (0.66–1.03)^#^	21.2 (393)	43.0 (795)	Ref
Female	37.3 (558)	Ref	13.9 (207)	48.8 (729)	**1.41 (1.15–1.75)** ^**^
** Age**					
40–49 years	39.4 (121)	1.55 (0.99–2.42)^#^	12.1 (37)	48.5 (149)	1.34 (0.87–2.06)
50–59 years	35.2 (249)	1.02 (0.75–1.39)	16.8 (119)	48.0 (340)	1.16 (0.87–1.55)
60–69 years	35.0 (454)	0.84 (0.66–1.07)	19.0 (246)	46.0 (596)	0.97 (0.77–1.21)
70 years and older	38.1 (328)	Ref	19.1 (164)	42.8 (368)	Ref
** Educational level**					
Lower	37.8 (335)	**1.37 (1.05–1.77)** ^*^	18.7 (166)	43.52 (386)	1.05 (0.82–1.35)
Medium	35.8 (361)	1.16 (0.90–1.49)	17.7 (178)	46.5 (469)	1.06 (0.84–1.34)
Higher	36.2 (524)	Ref	17.7 (256)	46.2 (669)	Ref
** Urbanization**					
Rural areas	33.8 (306)	Ref	16.8 (152)	49.3 (446)	**1.46 (1.10–1.94)** ^*^
Hardly urbanized areas	38.2 (301)	1.14 (0.85–1.53)	18.1 (143)	43.7 (344)	1.22 (0.91–1.63)
Moderately urbanized areas	37.1 (236)	0.97 (0.72–1.32)	21.5 (137)	41.4 (263)	Ref
Strongly or extremely urbanized areas	35.7 (301)	1.31 (0.99–1.74) ^#^	16.9 (143)	47.4 (400)	**1.50 (1.13–1.98)** ^**^
**Number of emotional supporters**					
** Sex**					
Male	46.9 (868)	1.27 (1.00-1.63)^#^	17.2 (318)	35.9 (664)	1.10 (0.86–1.40)
Female	50.1 (749)	Ref	12.9 (193)	36.9 (552)	Ref
** Age**					
40–49 years	48.2 (148)	0.94 (0.59–1.47)	13.0 (40)	38.8 (119)	1.03 (0.65–1.62)
50–59 years	47.2 (334)	0.98 (0.70–1.37)	13.0 (92)	39.8 (282)	1.23 (0.88–1.73)
60–69 years	49.5 (641)	1.05 (0.81–1.38)	15.2 (197)	35.3 (458)	1.04 (0.79–1.36)
70 years and older	46.7 (402)	Ref	17.8 (153)	35.5 (305)	Ref
** Educational level**					
Low	46.0 (408)	1.24 (0.93–1.65)	18.8 (167)	35.2 (312)	0.96 (0.73–1.28)
Medium	45.9 (463)	0.99 (0.75–1.31)	15.5 (156)	38.6 (389)	1.00 (0.76–1.32)
High	51.5 (746)	Ref	13.0 (188)	35.5 (515)	Ref
** Urbanization**					
Rural areas	48.1 (435)	Ref	13.9 (126)	37.9 (343)	**1.42 (1.01-2.00)** ^*^
Hardly urbanized areas	46.4 (366)	0.87 (0.63–1.22)	15.4 (121)	38.2 (301)	1.26 (0.90–1.78)
Moderately urbanized areas	50.8 (323)	0.95 (0.68–1.34)	14.8 (94)	34.4 (219)	Ref
Strongly or extremely urbanized areas	49.5 (418)	1.10 (0.81–1.50)	15.6 (132)	34.8 (294)	1.17 (0.85–1.62)
**Number of practical supporters**					
** Sex**					
Male	41.6 (770)	**1.49 (1.24–1.78)** ^***^	30.5 (564)	27.9 (516)	1.17 (0.97–1.41)
Female	38.5 (575)	Ref	32.4 (484)	29.1 (435)	Ref
** Age**					
40–49 years	44.6 (137)	**1.46 (1.03–2.08)** ^*^	25.1 (77)	30.3 (93)	1.30 (0.90–1.89)
50–59 years	40.3 (285)	1.00 (0.78–1.28)	32.2 (228)	27.5 (195)	0.92 (0.71–1.20)
60–69 years	38.9 (504)	0.92 (0.75–1.12)	33.4 (433)	27.7 (359)	0.88 (0.71–1.08)
70 years and older	42.4 (365)	Ref	30.5 (262)	27.1 (233)	Ref
** Educational level**					
Low	37.2 (330)	1.08 (0.88–1.34)	32.8 (291)	30.0 (266)	1.11 (0.89–1.39)
Medium	40.1 (404)	1.02 (0.84–1.25)	32.0 (323)	27.9 (281)	0.99 (0.80–1.23)
High	42.2 (611)	Ref	30.0 (434)	27.9 (404)	Ref
** Urbanization**					
Rural areas	40.7 (368)	Ref	28.0 (253)	31.3 (283)	**1.35 (1.06–1.70)** ^*^
Hardly urbanized areas	43.5 (343)	1.05 (0.83–1.33)	30.2 (238)	26.3 (207)	1.04 (0.82–1.34)
Moderately urbanized areas	37.4 (238)	0.82 (0.64–1.06)	34.0 (216)	28.6 (182)	1.02 (0.79–1.32)
Strongly or extremely urbanized areas	39.9 (337)	0.90 (0.72–1.13)	32.6 (275)	27.5 (232)	Ref

All analyses were adjusted for network size and living arrangement in 2019

^a^OR (Odds Ratio), 95% CI (95% Confidence Interval)

^*^p < 0.05,

^**^p < 0.01,

^***^p < 0.001

### Functional social network characteristics

#### Changes in the number of supporters in sociodemographic subgroups

Changes in the number of supporters in sociodemographic subgroups are shown in Table 2. In short, in all groups, the number of informational supporters increased, and the number of emotional and practical supporters decreased. Still, the increase in informational supporters was most notable among women, people below 70 years of age, medium or highly educated persons, or living in rural areas. The decrease in emotional supporters was most prominent in women, older, and, highly educated persons. The decrease in practical supporters was most prominent in men.

Men, older, and lower educated persons had fewer informational and emotional supporters in 2020 compared to their counterparts. Also, persons living in more urbanized areas had fewer informational and practical supporters in 2020 (Table 3).

#### Individual level changes in social support

While in all sociodemographic subgroups the mean number of informational supporters increased, there was individual variation. Of the respondents, 36% (*n* = 1,220) had decreased their number of informational supporters (mean − 3 members, range of -33 to -1). In 18% of the respondents (*n* = 600), the number of informational supporters remained unchanged and in 46% of the respondents this number had increased (mean increase of 4 network members, range of 1 to 29). Compared to having an unchanged number of informational supporters, to have decreased these numbers was more likely when having a lower educational level, and to have increased numbers was associated when being a woman or living in rural or strongly/extremely urbanized areas (Table 5). All odds ratios ranged between 1.37 and 1.50.

For about half of the respondents (52%, *n* = 1,738), the number of emotional supporters had decreased (mean decrease of 5 members, range of -33 to -1). For 13% of the respondents (*n* = 434), the number of emotional supporters remained unchanged and for 35% of the respondents, the number increased (mean increase of 4 network members, range of 1 to 26). Compared to having an unchanged number of emotional supporters, to have increased these numbers was more likely when living in rural areas (OR: 1.42) (Table 5).

Of the respondents, 40% (*n* = 1,345) had decreased the number of practical supporters (mean decrease of 2 members, range of -33 to -1). For 31% of the respondents (*n* = 1048), the number of practical supporters remained unchanged. In 28% of the respondents, this number had increased (mean increase of 2 members, range of 1 to 14). Compared to having an unchanged number of practical supporters, to have decreased these numbers was more likely when being man, or younger; to have increased these numbers was more likely when living in rural areas (Table 5). All odds ratios ranged between 1.35 and 1.49.

## Discussion

This prospective cohort study assessed changes during the COVID-19 pandemic in social network structure (network size) and social network function (social support) in people from various sociodemographic subgroups, aged 40 years and older. In all subgroups, the average number of network members (network size) and the number of emotional and practical supporters had decreased. However, the average number of informational supporters had increased. Within the population, individual variation was observed, in that some individuals increased network sizes or the number of supporters: women, higher-educated persons, and persons living in rural areas. Those who were more likely to demonstrate decreases were men, lower-educated persons, and persons living in moderately, strongly, and extremely urbanized areas. Findings highlight the importance to evaluate social networks by both structure and function and account for sociodemographic subgroups.

In line with the reported loss of daily social contacts as a result of the implemented preventive measures, such as social distancing [5, 36], the current study demonstrated decreases in network size, including losses in the number of family members, friends, and others. Family contacts showed the smallest reduction, and therefore social networks became more family-centered during the pandemic. Family members are considered as key important relationship types (considered as strong ties) for support [37]; people often depend on family for informal care or as practical supporters. During a pandemic, individuals need social support, such as that provided by family members, but also by friends or neighbors [38]. However, with social distancing as the norm, friends might not have been ‘able’ to provide social support in physical proximity.

Social networks can act as a buffer in dealing with stressful life events including the COVID-19 pandemic by providing social support [39]. The results of the current study showed an overall increase in informational supporters, even though total network size decreased. This suggests that network members who did not provide social support, were not mentioned as important network members during the pandemic. Network members who were mentioned compensated the social support roles. Social support roles likely were attributed more centrally in the network. Family members likely fulfilled additional support roles, such as the provision of emotional support, since the current study showed an increase in family members providing emotional support, while numbers of non-family emotional and practical supporters had decreased (data not shown). These findings suggest that the functional (support) roles of existing network members may have changed.

Notably, the average number of informational supporters increased and thereby the percentage of network member who provided informational support, regardless of whether networks members were family, friends, or acquaintances. During a pandemic, people desire informational support [40, 41] and they might be able to generate support from existing network members or gaining new network members and mobilizing those members for social support. More distant members (so called weak ties), such as acquaintances are known to play an important role in the provision of informational support, especially new information, but are also important for expanding social networks through connections to other ties [16]. These new social relationships could then be harvested for social support. As this study demonstrated, some people were able to increase their network and gain new network members in times of adversity and uncertainty when social distancing was the norm. This may occur by digital contact for example. Digital contact might generate opportunities to contact network members who were previously contacted in person [42] or to contact new network members. However, future studies should assess whether in person contact could effectively be substituted by digital contact during stressful life events such as a pandemic.

Men, older, lower-educated persons, and people living in more urbanized areas were more likely to have decreased in number of network members, informational and practical supporters. Previous studies have shown that men and lower-educated persons receive less social support, are less satisfied with received support and experience more social strain [15, 30]. Moreover, a study among American and British adults also showed that men and lower-educated persons are less aware of the (health) benefits of social ties [43]. Individuals who are less aware of the benefits may be more vulnerable in managing their social networks for maintaining and gaining support.

Furthermore, in our current study, younger persons (40–50 years old) were more likely to have decreased the number of practical supporters. This is most likely a direct impact of social distancing measures, as practical support often requires in-person contact. Practical support might have been postponed or canceled by this age group, whereas older and more vulnerable persons became more reliant on others, especially on members, for help with groceries or tasks in or around the house.

Notably, various types of individual people demonstrated increases in network size and supporters i.e., they were more likely to be women, higher-educated, or living in rural areas, and these characteristics were also associated with having larger social networks, and more informational and emotional supporters during the COVID-19 pandemic. Previous study demonstrated that, during the COVID-19 pandemic, women’s contact with their social relationships intensified and they strengthened their networks [44]. In addition, previous studies reported higher levels of perceived support after stressful life-events among higher educated persons [45], suggesting that they have increased access to social support [39] by attributing especially informational support roles more centrally in the network.

Strengths should be acknowledged. First, a strength is the large study cohort and the longitudinal study design which provides the opportunity to analyze changes in social network characteristics between 2019 (pre-pandemic) and 2020 (after the first wave of the pandemic during the COVID-19 pandemic). Second, to measure social networks, a name generator questionnaire was used, which is a reliable and valid method for measuring social networks. It needs to be acknowledged that the current study focused on independently living adults. Some limitations should be noted as well. First, the current study design is unable to provide prove whether changes in social network structure and function occurred because of the pandemic as the study naturally did not include a control population [one who had not experienced the pandemic]. Secondly, we were unable to measure network turnover (e.g., the expansion or shrinkage of close social network members) [31] as it was unknown whether network members in 2020 were the same or different than in 2019 per respondent (such types of data were not collected). Network turnover may impact one’s health, and the magnitude of this impact depends on the type of relationship gained or lost (e.g., strength of relationships, provision of social support or social strain, or a deliberate change). Third, participants lost to follow-up were slightly younger than respondents, although this did not affect results, as we stratified by age-groups.

## Conclusion

This study emphasizes the importance of evaluating both structure and function of social networks when the goal is to assess impact of the COVID-19 pandemic on people’s social relationships. Social networks can act as a buffer for the impact of the COVID-19 pandemic. Results showed that although network structure and function changed for all sociodemographic subgroups, certain some individuals managed to reorganize their social networks, by attributing social support roles more centrally. Some sociodemographic subgroups were more likely to decrease in social network structure and function, enhancing already existing health inequalities.

## Supplementary Information

**Additional file** **1: **Name generator questionnaire. Name generator items used in the baseline and follow-up questionnaire to assess social networks.

**Additional file** **2: **Demographic characteristics of people aged 40 years and older in the Netherlands (SaNAE study population) per change in network size or support.

## Data Availability

The dataset supporting the conclusions of this article is available upon request. Data contains potentially identifying and sensitive information of respondents. Due to the General Data Protection Regulation, it is not allowed to distribute or share any personal data that can be traced back (direct or indirect) to an individual. Moreover, publicly sharing the data would not be in accordance with participant consent for this study. Therefore, interested researchers should contact the head of the data-archiving of the Public Health Service South Limburg (Helen Sijstermans: helen.sijstermans@ggdzl.nl) when they would like to re-use data.

## References

[1] Advice for the public. https://www.who.int/emergencies/diseases/novel-coronavirus-2019/advice-for-public. Accessed 14 Oct 2021.

[2] Feehan DM, Mahmud AS (2021). Quantifying population contact patterns in the United States during the COVID-19 pandemic. Nat Commun 2021 121.

[3] Auranen K, Shubin M, Karhunen M, Sivelä J, Leino T, Nurhonen M (2021). Social Distancing and SARS-CoV-2 Transmission Potential Early in the Epidemic in Finland. Epidemiology.

[4] Latsuzbaia A, Herold M, Bertemes J-P, Mossong J (2020). Evolving social contact patterns during the COVID-19 crisis in Luxembourg. PLoS ONE.

[5] Jarvis CI, Van Zandvoort K, Gimma A, Prem K, Klepac P, Rubin GJ (2020). Quantifying the impact of physical distance measures on the transmission of COVID-19 in the UK. BMC Med..

[6] Howick J, Kelly P, Kelly M. Establishing a causal link between social relationships and health using the Bradford Hill Guidelines. SSM - Popul Heal. 2019. 10.1016/j.ssmph.2019.100402. 10.1016/j.ssmph.2019.100402 PMC6527915 31193417

[7] Coleman ME, Manchella MK, Roth AR, Peng S, Perry BL (2022). What kinds of social networks protect older adults’ health during a pandemic? The tradeoff between preventing infection and promoting mental health. Soc Networks.

[8] Armitage R, Nellums LB (2020). COVID-19 and the consequences of isolating the elderly. The Lancet Public Health.

[9] Yang YC, Li T, Frenk SM (2014). Social Network Ties and Inflammation in U.S. Adults with Cancer. Biodemography Soc Biol.

[10] Eng P, Rimm E, Fitzmaurice G, Kawachi I (2002). Social ties and change in social ties in relation to subsequent total and cause-specific mortality and coronary heart disease incidence in men. Am J Epidemiol.

[11] Brinkhues S, Dukers-Muijrers NHTM, Hoebe CJPA, Van Der Kallen CJH, Koster A, Henry RMA (2018). Social network characteristics are associated with type 2 diabetes complications: The Maastricht study. Diabetes Care.

[12] Valente TW. Social networks and health behavior. In: In: B. Rimer, K. Glanz, V. Vishwanath, editor. Health behavior: Theory, Research & Practice. 6th edition. New York: Wiley; 2015. p. 205–22.

[13] Hakulinen C, Pulkki-Råback L, Markus Jokela, Ferrie JE, Aalto AM, Virtanen M (2016). Structural and functional aspects of social support as predictors of mental and physical health trajectories: Whitehall II cohort study. J Epidemiol Community Health.

[14] Berkman LF, Glass T, Berkman L, Kawachi I (2000). Social integration, social networks, social support and health. Social epidemiology.

[15] Antonucci TC, Akiyama H (1987). An examination of sex differences in social support among older men and women. Sex Roles 1987 1711.

[16] Granovetter MS (1973). The Strength of Weak Ties. Am J Sociol.

[17] Ajrouch KJ, Blandon AY, Antonucci TC (2005). Social Networks Among Men and Women: The Effects of Age and Socioeconomic Status. Journals Gerontol Ser B.

[18] Carstensen LL. Motivation for social contact across the life span: A theory of socioemotional selectivity. In: Nebraska Symposium on Motivation, 1992: Developmental perspectives on motivation. Lincoln, NE, US: University of Nebraska Press; 1993. p. 209–54. 1340521

[19] Carstensen LL, Fung HH, Charles ST (2003). Socioemotional Selectivity Theory and the Regulation of Emotion in the Second Half of Life. Motiv Emot.

[20] Fiori KL, Antonucci TC, Cortina KS (2006). Social network typologies and mental health among older adults. J Gerontol B Psychol Sci Soc Sci.

[21] Völker B (2023). Networks in lockdown: The consequences of COVID-19 for social relationships and feelings of loneliness. Soc Networks.

[22] Verity R, Okell LC, Dorigatti I, Winskill P, Whittaker C, Imai N (2020). Estimates of the severity of coronavirus disease 2019: a model-based analysis. Lancet Infect Dis.

[23] Figliozzi S, Masci PG, Ahmadi N, Tondi L, Koutli E, Aimo A (2020). Predictors of adverse prognosis in COVID-19: A systematic review and meta-analysis. Eur J Clin Invest.

[24] Ahrenfeldt LJ, Otavova M, Christensen K, Lindahl-Jacobsen R (2021). Sex and age differences in COVID-19 mortality in Europe. Wien Klin Wochenschr.

[25] Nielsen J, Nørgaard SK, Lanzieri G, Vestergaard LS, Moelbak K (2021). Sex-differences in COVID-19 associated excess mortality is not exceptional for the COVID-19 pandemic. Sci Reports.

[26] Vahidy FS, Pan AP, Ahnstedt H, Munshi Y, Choi HA, Tiruneh Y (2021). Sex differences in susceptibility, severity, and outcomes of coronavirus disease 2019: Cross-sectional analysis from a diverse US metropolitan area. PLoS ONE.

[27] Cuadros DF, Branscum AJ, Mukandavire Z, Miller FDW, MacKinnon N (2021). Dynamics of the COVID-19 epidemic in urban and rural areas in the United States. Ann Epidemiol.

[28] Mishra V, Seyedzenouzi G, Almohtadi A, Chowdhury T, Khashkhusha A, Axiaq A (2021). Health Inequalities During COVID-19 and Their Effects on Morbidity and Mortality. J Healthc Leadersh.

[29] Boterman WR (2020). Urban-Rural Polarisation in Times of the Corona Outbreak? The Early Demographic and Geographic Patterns of the SARS-CoV-2 Epidemic in the Netherlands. Tijdschr Econ Soc Geogr.

[30] Weyers S, Dragano N, Möbus S, Beck EM, Stang A, Möhlenkamp S (2008). Low socio-economic position is associated with poor social networks and social support: results from the Heinz Nixdorf Recall Study. Int J Equity Health.

[31] Cornwell B (2015). Social Disadvantage and Network Turnover. Journals Gerontol Ser B.

[32] von Elm E, Altman DG, Egger M, Pocock SJ, Gøtzsche PC, Vandenbroucke JP (2008). The Strengthening the Reporting of Observational Studies in Epidemiology (STROBE) statement: guidelines for reporting observational studies. J Clin Epidemiol.

[33] GGD Gezondheidsmonitor Volwassenen en Ouderen. 2020. 2021. https://www.ggdzl.nl/professionals/publicaties/factsheets-en-rapporten/. Accessed 22 Feb 2022.

[34] den Dulk C, van de Stadt H, Vliegen J (1992). Een nieuwe maatstaf voor stedelijkheid: de omgevingsadressendichtheid. Maandstat van Bevolk.

[35] Steijvers LCJ, Brinkhues S, Hoebe CJPA, van Tilburg TG, Claessen V, Bouwmeester-Vincken N (2021). Social networks and infectious diseases prevention behavior: A cross-sectional study in people aged 40 years and older. PLoS ONE.

[36] Tomori DV, Rübsamen N, Berger T, Scholz S, Walde J, Wittenberg I (2021). Individual social contact data and population mobility data as early markers of SARS-CoV-2 transmission dynamics during the first wave in Germany—an analysis based on the COVIMOD study. BMC Med.

[37] Hill RA, Dunbar RIM (2003). Social network size in humans. Hum Nat 2003 141.

[38] Li F, Luo S, Mu W, Li Y, Ye L, Zheng X (2021). Effects of sources of social support and resilience on the mental health of different age groups during the COVID-19 pandemic. BMC Psychiatry.

[39] Nitschke JP, Forbes PAG, Ali N, Cutler J, Apps MAJ, Lockwood PL (2021). Resilience during uncertainty? Greater social connectedness during COVID-19 lockdown is associated with reduced distress and fatigue. Br J Health Psychol.

[40] Comfort LK, Ko K, Zagorecki A. Coordination in Rapidly Evolving Disaster Response Systems: The Role of Information. http://dx.doi.org.mu.idm.oclc.org/101177/0002764204268987. 2016;48:295–313.

[41] Kalgotra P, Gupta A, Sharda R (2021). Pandemic information support lifecycle: Evidence from the evolution of mobile apps during COVID-19. J Bus Res.

[42] Quan-Haase A, Mo GY, Wellman B. Connected seniors: how older adults in East York exchange social support online and offline. 10.1080/1369118X.2017.1305428. 2017;20:967–83.

[43] Haslam SA, McMahon C, Cruwys T, Haslam C, Jetten J, Steffens NK (2018). Social cure, what social cure? The propensity to underestimate the importance of social factors for health. Soc Sci Med.

[44] Reisch T, Heiler G, Hurt J, Klimek P, Hanbury A, Thurner S (2021). Behavioral gender differences are reinforced during the COVID-19 crisis. Sci Reports.

[45] Kaniasty K (2012). Predicting social psychological well-being following trauma: The role of postdisaster social support. Psychol Trauma Theory Res Pract Policy.

