# Supplementary material for: Changes in structure and function of social networks of independently living middle-aged and older adults in diverse sociodemographic subgroups during the COVID-19 pandemic: a longitudinal study
Source: BMC Public Health. 2022 Dec 3;22:2253. doi: 10.1186/s12889-022-14500-2 (PMC9719122; doi:10.1186/s12889-022-14500-2)
Supplement: Supplementary file 1 — Additional file 1: Name generator questionnaire. Name generator items used in the baseline and follow-up questionnaire to assess social networks. [file 12889_2022_14500_MOESM1_ESM.docx]

**Supplementary file 1. Name generator questionnaire**

**Social networks**

The following questions are about people who are important in your life, who provide support or to whom you provide social support. A reliant method is used to identify and measure your social network. For each question, you can provide the first name and first letter of the last name of the most important persons. The names are only used to fill in the questionnaire and to measure the social network. Researchers are unable to identify or reach out to these persons.

Family members who are important in my life currently, who provide social support (emotional, informational, or practical) or to whom I provide support, are:

List the first name and the first letter of the last name of your most important family members

1. ____
2. ____
3. ____
4. ____
5. ____
6. ____
7. ____
8. ____
9. ____
10. ____
11. ____
12. ____
13. ____
14. ____
15. ____
16. I do not have any family members who are important to me or provide social support.

Friends who are important in my life currently, who provide social support (emotional, informational, or practical) or to whom I provide support, are:

List the first name and the first letter of the last name of your most important friends

1. ____
2. ____
3. ____
4. ____
5. ____
6. ____
7. ____
8. ____
9. ____
10. ____
11. I do not have any friends who are important to me or provide social support.

Colleagues, acquaintances, or neighbors who are important in my life currently, who provide social support (emotional, informational, or practical) or to whom I provide support, are:

List the first name and the first letter of the last name of your most important colleagues, acquaintances, or neighbors

1. ____
2. ____
3. ____
4. ____
5. ____
6. ____
7. ____
8. ____
9. ____
10. ____
11. I do not have any colleagues, acquaintances or neighbors who are important to me or provide social support.

These persons are important to me, but I have not mentioned them before:

1. ____
2. ____
3. ____
4. ____
5. ____
6. I have mentioned all persons who are important to me in the previous questions.

In the previous questions, you have mentioned several persons. We would like to know who provides social support and what kind of support.

Multiple answers possible

|  | Gives advice if I have a problem | Helps me with jobs in or around the house (or groceries) | Talk about important topics | Talk about health status | Provides another type of support | Conviviality | I receive care (general practitioner, nurse, etc.) | I do not receive support from this person | I take care of this person |
| --- | --- | --- | --- | --- | --- | --- | --- | --- | --- |
| [Name family member 1] |  |  |  |  |  |  |  |  |  |
| [Name family member 2] |  |  |  |  |  |  |  |  |  |
| [Name family member 3] |  |  |  |  |  |  |  |  |  |
| [Name family member 4] |  |  |  |  |  |  |  |  |  |
| [Name family member 5] |  |  |  |  |  |  |  |  |  |
| [Name family member 6] |  |  |  |  |  |  |  |  |  |
| [Name family member 7] |  |  |  |  |  |  |  |  |  |
| [Name family member 8] |  |  |  |  |  |  |  |  |  |
| [Name family member 9] |  |  |  |  |  |  |  |  |  |
| [Name family member 10] |  |  |  |  |  |  |  |  |  |
| [Name family member 11] |  |  |  |  |  |  |  |  |  |
| [Name family member 12] |  |  |  |  |  |  |  |  |  |
| [Name family member 13] |  |  |  |  |  |  |  |  |  |
| [Name family member 14] |  |  |  |  |  |  |  |  |  |
| [Name family member 15] |  |  |  |  |  |  |  |  |  |
| [Name friend 1] |  |  |  |  |  |  |  |  |  |
| [Name friend 2] |  |  |  |  |  |  |  |  |  |
| [Name friend 3] |  |  |  |  |  |  |  |  |  |
| [Name friend 4] |  |  |  |  |  |  |  |  |  |
| [Name friend 5] |  |  |  |  |  |  |  |  |  |
| [Name friend 6] |  |  |  |  |  |  |  |  |  |
| [Name friend 7] |  |  |  |  |  |  |  |  |  |
| [Name friend 8] |  |  |  |  |  |  |  |  |  |
| [Name friend 9] |  |  |  |  |  |  |  |  |  |
| [Name friend 10] |  |  |  |  |  |  |  |  |  |
| [Name acquaintance 1] |  |  |  |  |  |  |  |  |  |
| [Name acquaintance 2] |  |  |  |  |  |  |  |  |  |
| [Name acquaintance 3] |  |  |  |  |  |  |  |  |  |
| [Name acquaintance 4] |  |  |  |  |  |  |  |  |  |
| [Name acquaintance 5] |  |  |  |  |  |  |  |  |  |
| [Name acquaintance 6] |  |  |  |  |  |  |  |  |  |
| [Name acquaintance 7] |  |  |  |  |  |  |  |  |  |
| [Name acquaintance 8] |  |  |  |  |  |  |  |  |  |
| [Name acquaintance 9] |  |  |  |  |  |  |  |  |  |
| [Name acquaintance 10] |  |  |  |  |  |  |  |  |  |
| [Name extra 1] |  |  |  |  |  |  |  |  |  |
| [Name extra 2] |  |  |  |  |  |  |  |  |  |
| [Name extra 3] |  |  |  |  |  |  |  |  |  |
| [Name extra 4] |  |  |  |  |  |  |  |  |  |
| [Name extra 5] |  |  |  |  |  |  |  |  |  |
