# Supplementary material for: Changes in structure and function of social networks of independently living middle-aged and older adults in diverse sociodemographic subgroups during the COVID-19 pandemic: a longitudinal study
Source: BMC Public Health. 2022 Dec 3;22:2253. doi: 10.1186/s12889-022-14500-2 (PMC9719122; doi:10.1186/s12889-022-14500-2)
Supplement: Supplementary file 2 — Additional file 2: Demographic characteristics of people aged 40 years and older in the Netherlands (SaNAE study population) per change in network size or support. [file 12889_2022_14500_MOESM2_ESM.docx]

|  | **Network size** | | | **# of informational supporters** | | | **# of emotional supporters** | | | **# of practical supporters** | | |
| --- | --- | --- | --- | --- | --- | --- | --- | --- | --- | --- | --- | --- |
|  | Decrease (n=1545) | Stable (n=861) | Increase (n=938) | Decrease (n=1220) | Stable (n=600) | Increase (n=1524) | Decrease (n=1738) | Stable (n=434) | Increase (n=1172) | Decrease (n=1345) | Stable (n=1048) | Increase (n=951) |
|  | % (n) | % (n) | % (n) | % (n) | % (n) | % (n) | % (n) | % (n) | % (n) | % (n) | % (n) | % (n) |
| **Sex** |  |  |  |  |  |  |  |  |  |  |  |  |
| Male | 54.8 (847) | 58.4 (503) | 53.3 (500) | 54.3 (662) | 65.5 (393) | 52.2 (795) | 53.8 (935) | 60.1 (261) | 55.8 (654) | 57.2 (770) | 53.8 (564) | 54.3 (516) |
| Female | 45.2 (698) | 41.6 (358) | 46.7 (438) | 45.7 (558) | 34.5 (207) | 47.8 (729) | 46.2 (803) | 39.9 (173) | 44.2 (518) | 42.8 (575) | 46.2 (484) | 45.7 (435) |
| **Age** |  |  |  |  |  |  |  |  |  |  |  |  |
| 40-49 years | 7.1 (110) | 7.0 (60) | 9.8 (92) | 8.9 (108) | 5.7 (34) | 7.9 (120) | 7.6 (132) | 8.3 (36) | 8.0 (94) | 8.6 (116) | 6.5 (68) | 8.2 (78) |
| 50-59 years | 17.2 (265) | 20.8 (179) | 21.4 (201) | 18.6 (227) | 17.2 (103) | 20.7 (315) | 18.4 (319) | 18.0 (78) | 21.2 (248) | 19.5 (262) | 19.7 (206) | 18.6 (177) |
| 60-69 years | 37.9 (585) | 39.6 (341) | 37.7 (354) | 35.8 (437) | 40.7 (244) | 39.3 (599) | 39.1 (680) | 37.3 (162) | 37.4 (438) | 37.5 (504) | 40.1 (420) | 37.4 (356) |
| 70 years or older | 37.9 (585) | 32.6 (281) | 31.0 (291) | 36.7 (448) | 36.5 (219) | 32.2 (490) | 34.9 (607) | 36.4 (158) | 33.4 (392) | 34.4 (463) | 33.8 (354) | 35.8 (340) |
| **Educational level** |  |  |  |  |  |  |  |  |  |  |  |  |
| Low | 27.1 (418) | 29.3 (252) | 23.1 (217) | 27.5 (335) | 27.7 (166) | 25.3 (386) | 25.9 (450) | 30.0 (130) | 26.2 (307) | 24.5 (330) | 27.8 (291) | 28.0 (266) |
| Medium | 29.2 (451) | 32.2 (277) | 29.9 (280) | 29.6 (361) | 29.7 (178) | 30.8 (469) | 28.7 (499) | 31.1 (135) | 31.9 (374) | 30.0 (404) | 30.8 (323) | 29.5 (281) |
| High | 43.8 (676) | 38.6 (332) | 47.0 (441) | 43.0 (524) | 42.7 (256) | 43.9 (669) | 45.4 (789) | 38.9 (169) | 41.9 (491) | 45.4 (611) | 41.4 (434) | 42.5 (404) |
| **Urbanization** |  |  |  |  |  |  |  |  |  |  |  |  |
| Rural areas | 25.0 (386) | 26.8 (231) | 30.6 (287) | 25.1 (306) | 25.3 (152) | 29.3 (446) | 26.6 (463) | 24.0 (104) | 28.8 (337) | 27.4 (368) | 24.1 (253) | 29.8 (283) |
| Hardly urbanized areas | 24.1 (372) | 23.2 (200) | 23.0 (216) | 24.7 (301) | 23.8 (143) | 22.6 (344) | 22.8 (396) | 23.7 (103) | 24.7 (289) | 25.5 (343 | 22.7 (238) | 21.8 (207) |
| Moderately urbanized areas | 20.2 (312) | 18.5 (159) | 17.6 (165) | 19.3 (236) | 22.8 (137) | 17.3 (263) | 19.4 (338) | 21.2 (92) | 17.6 (206) | 17.7 (238) | 20.6 (216) | 19.1 (182) |
| Strongly or extremely urbanized areas | 30.7 (475) | 31.5 (271) | 28.8 (270) | 30.9 (377) | 28.0 (168) | 30.9 (471) | 31.1 (541) | 31.1 (135) | 29.0 (340) | 29.4 (396) | 32.5 (341) | 29.3 (279) |

# Supplementary table 1. Demographic characteristics of people aged 40 years and older in the Netherlands (SaNAE study population) per change in network size or support
